# Divergence of ecosystem services in U.S. National Forests and Grasslands under a changing climate

**DOI:** 10.1038/srep24441

**Published:** 2016-04-21

**Authors:** Kai Duan, Ge Sun, Shanlei Sun, Peter V. Caldwell, Erika C. Cohen, Steven G. McNulty, Heather D. Aldridge, Yang Zhang

**Affiliations:** 1 Department of Marine, Earth, and Atmospheric Sciences, North Carolina State University, Raleigh, NC, USA; 2 Eastern Forest Environmental Threat Assessment Center, USDA Forest Service, Raleigh, NC, USA; 3 Key Laboratory of Meteorological Disaster of Ministry of Education, Nanjing University of Information Science & Technology, Nanjing, Jiangsu, China; 4 Coweeta Hydrologic Laboratory, USDA Forest Service, Otto, NC, USA; 5 State Climate Office of North Carolina, North Carolina State University, Raleigh, NC, USA

## Abstract

The 170 National Forests and Grasslands (NFs) in the conterminous United States are public lands that provide important ecosystem services such as clean water and timber supply to the American people. This study investigates the potential impacts of climate change on two key ecosystem functions (i.e., water yield and ecosystem productivity) using the most recent climate projections derived from 20 Global Climate Models (GCMs) of the Coupled Model Intercomparison Project phase 5 (CMIP5). We find that future climate change may result in a significant reduction in water yield but an increase in ecosystem productivity in NFs. On average, gross ecosystem productivity is projected to increase by 76 ~ 229 g C m^−2^ yr^−1^ (8% ~ 24%) while water yield is projected to decrease by 18 ~ 31 mm yr^−1^ (4% ~ 7%) by 2100 as a result of the combination of increased air temperature (+1.8 ~ +5.2 °C) and precipitation (+17 ~ +51 mm yr^−1^). The notable divergence in ecosystem services of water supply and carbon sequestration is expected to intensify under higher greenhouse gas emission and associated climate change in the future, posing greater challenges to managing NFs for both ecosystem services.

Forests and grasslands are increasingly recognized for their function in providing ecosystem services, such as clean water supply, carbon sequestration, and biodiversity conservation^1^,^2^,^3^. Forests enhance watershed stability and resilience by absorbing rainfall, cleansing water, mitigating floods, and providing habitat for wildlife^4^. Forests are also a large carbon sink and play an increasing role in mitigating global warming^5^. In the U.S., forested land covers about 29% of the country’s land surface, but supplies over half of the total fresh water^6^ and offset up to 10% ~ 20% of the current fossil fuel emissions^7^. The U.S. National Forests and Grasslands System (NFs) was established as federal forest reserves in 1891 amid increasing public concern about land degradation^4^. The 781,000 km^2^ (193 million acres) NFs represent only 9% of the U.S. land area, but play an important role in providing freshwater (i.e., 14% national water supply), recreation, wildlife habitats, and other ecosystem services to the American public^8^. However, these services are vulnerable to multiple environmental threats including a changing climate^9^,^10^.

Watershed water yield (also referred to as runoff or streamflow), evapotranspiration (ET), gross ecosystem productivity (GEP), and net ecosystem exchange (NEE) are key indicators of forest and grassland ecosystem services^11^,^12^. Climate change significantly alters global patterns of ET^13^, river flow^14^, ecosystem productivity^15^ and carbon balances^16^, and consequently threatens local economy in the forestry industry^17^. According to the fifth Assessment Report (AR5) of the Intergovernmental Panel on Climate Change (IPCC), the past three decades were possibly the warmest 30-year period of the last 1,400 years in the Northern Hemisphere^18^. Such a rise in air temperature usually leads to an increase in potential evapotranspiration (PET), hence altering ecosystem ET, water yield, and carbon sequestration along with underlying surface perturbations and changes in regional moisture and heat budgets^15^,^19^. Climate change also impacts the magnitude and distribution of precipitation, increasing risks of droughts, floods, and other extreme hydroclimatic events^18^,^20^,^21^,^22^, that directly alter the regional water balance and long-term streamflow patterns^23^. These changes imply that water supply as well as energy and carbon fluxes may become more variable among terrestrial ecosystems with substantial uncertainties in the future. A better understanding of water and carbon responses to climate change is vital for land managers and decision makers to develop appropriate mitigation and adaptation strategies to sustain the broad variety of ecosystem services^24^.

This study investigates the water and carbon balances in 170 NFs (150 national forests and 20 national grasslands) across the conterminous United States (CONUS) (Fig. 1) under various climate scenarios using a consistent integrated modeling approach. Our main aim is to provide a comprehensive assessment of potential climate change impacts on key NF ecosystem services by quantifying water (i.e., ET and water yield) and carbon (i.e., GEP and NEE) responses to climate change at a fine spatial scale (i.e., 12-digit Hydrologic Unit Code watersheds). We integrate a regional ecohydrological modeling system and the most recent climate projections from 20 different Atmosphere-Ocean Global Climate Models (AOGCMs) of Coupled Model Intercomparison Project phase 5 (CMIP5), which have been statistically downscaled using the Multivariate Adaptive Constructed Analogs (MACA) method. The large set of GCM simulations represents a variety of the future climates and enables robust quantification of major uncertainties in future estimates of ecosystem responses.

## Results

We use a previously validated ecosystem accounting model, i.e., Water Supply Stress Index model (WaSSI)^25^,^26^, to simulate the water and carbon cycles driven by different climate scenarios. The projections for surface meteorological variables are derived from 20 GCMs for both historical climate forcings and two future Representative Concentration Pathways (RCPs) scenarios including RCP4.5 and RCP8.5, as representatives of the intermediate and high greenhouse gas (GHG) emission scenarios. Five 30-yr series of monthly precipitation and temperature are extracted to represent the current and future climates: (i) Baseline - baseline period in 1970–1999; (ii) RCP4.5/2030s - 1^st^ future period in 2020–2049 under RCP4.5; (iii) RCP4.5/2080s - 2^nd^ future period in 2070–2099 under RCP4.5; (iv) RCP8.5/2030s - 1^st^ future period in 2020–2049 under RCP8.5; (v) RCP8.5/2080s - 2^nd^ future period in 2070–2099 under RCP8.5. The changes in water and carbon components are evaluated by the differences between the baseline scenario and the four future scenarios. The projected changes in mean temperature over the CONUS vary between +0.8 °C and +7.0 °C among the climate models and scenarios. The median temperature rise of the inter-GCM ranges reaches 1.8 °C, 3.1 °C, 2.0 °C, and 5.2 °C in the four future scenarios, respectively (see Supplementary Table S1).

### Changes in water balance

**Figure 1:**
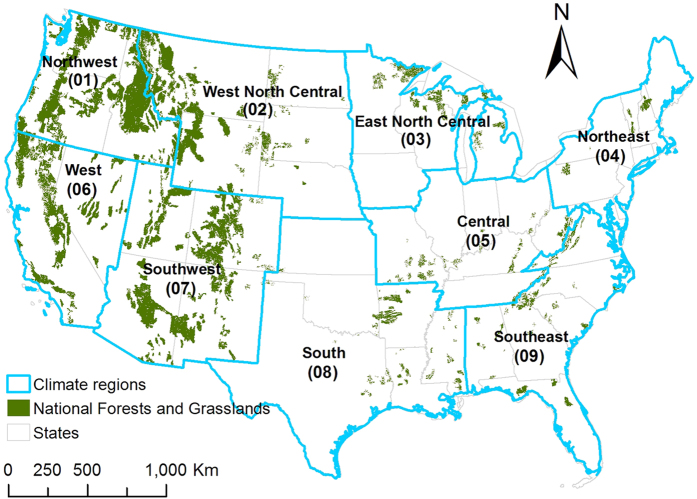
Spatial distribution of the 170 National Forests and Grasslands (NFs) and nine climate regions over the CONUS. Maps were generated using ArcGIS 10.0 (www.esri.com/software/arcgis).

**Figure 2:**
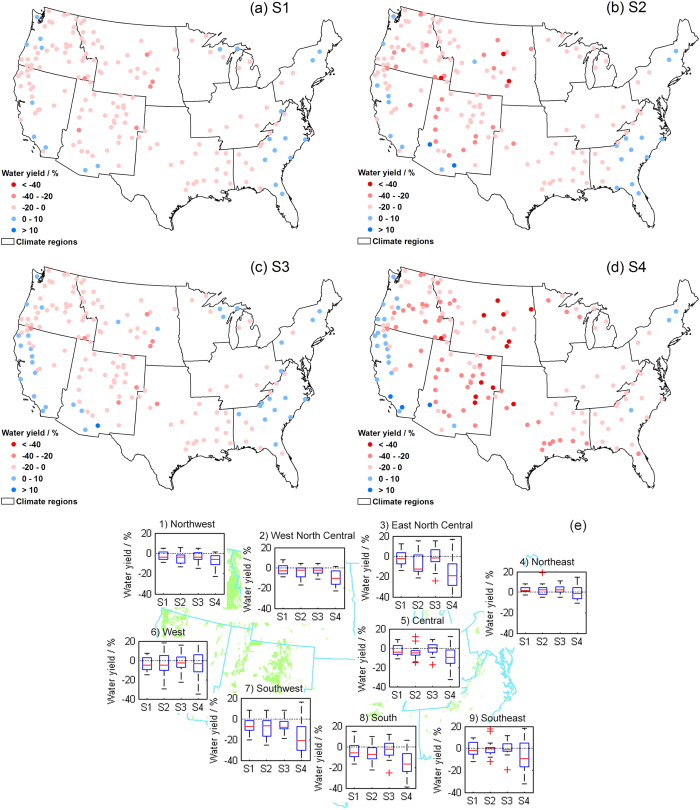
Changes in multi-year mean annual water yield (%) from the baseline (1970–1999) to the future periods (2020–2049 and 2070–2099) under RCP4.5 and RCP8.5 scenarios in the 170 NFs and nine climate regions. The blue and red dots (Fig. a–d) indicate positive and negative changes derived from the multi-model mean simulations of the 20 GCMs in each NF, respectively. The box-whisker plots (Fig. e) display the area-averaged changes projected from different GCMs in the nine climate regions. The four future scenarios for different time periods and greenhouse gas emission levels are denoted by S1 (RCP4.5/2030s), S2 (RCP4.5/2080s), S3 (RCP8.5/2030s), and S4 (RCP8.5/2080s) in the x-axis. The area-averaged values within each climate region are shown along the y-axis, and the results under different scenarios are listed along the x-axis. The boxes cover the ranges from the 25% quartile to the 75% quartile of the distributions (Inter-Quartile Range, IQR), with median values marked by red line within each box, and points outside the whiskers are taken as extreme outliers. Maps were generated using ArcGIS 10.0 (www.esri.com/software/arcgis).

Figure 2 presents the spatial and temporal distribution of projected changes in multi-year mean annual water yield at individual NFs and by climate region. For NFs, the multi-model average changes span between −30% ~ +8% (Fig. 2a) and −31% ~ +16% (Fig. 2c) for the years of 2030s, and between −45% ~ +15% (Fig. 2b) and −68% ~ +18% (Fig. 2d) for the years of 2080s. Decreasing signals for water yield are found in most NFs, and severe declines are most likely to occur across the Northwest, West North Central, and Southwest regions in the western CONUS (see the distribution of 170 NFs and nine climatic regions in Fig. 1). The exceptional cases with positive signals are most often found in four regions neighboring the oceans, including the coastal areas of the Northwest, West, Northeast and Southeast regions. More significant decreases in water yield are generally projected for inland watersheds than for coastal areas. The regional future changes in water yield (Fig. 2e) vary between −40% (decrease) and +20% (increase), reflecting the inconsistency of changing directions. Nevertheless, the median lines and the Inter-Quartile Ranges (IQRs) clearly suggest a decreasing trend with a high confidence. The median values stay on the negative side of the zero line in almost all the regions except the Northeast, where an increasing signal is found but it only contributes to 1% of the whole NF area. Generally, the vertical positions of the boxes suggest consistent changing trends, whereas the level of uncertainty varies with forecast time period and RCP scenarios. The GCMs show more agreement on the simulations in 2030s than in 2080s, indicating that the uncertainty increases dramatically over time under both RCPs. In particular, a much higher level of uncertainty can be observed in the RCP8.5/2080 scenario than other cases. Such results imply that there is still a large uncertainty involved in the projection of global change in the state-of-art GCMs, and the uncertainty becomes larger when predicting a longer term ecosystem response or at a higher emission level.

**Figure 3:**
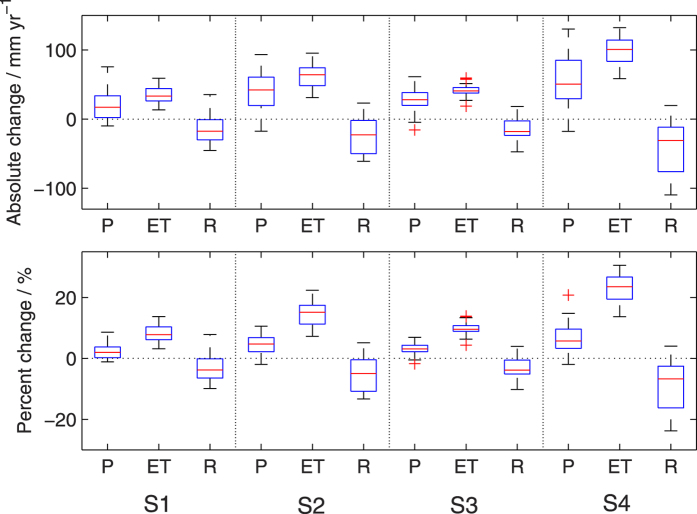
Changes in the total of mean annual precipitation (P), ET, and water yield (R) over the NF areas from the baseline to the future scenarios of S1 (RCP4.5/2030s), S2 (RCP4.5/2080s), S3 (RCP8.5/2030s), and S4 (RCP8.5/2080s).

The pattern of changes in water yield is controlled by the precipitation (P) participating between ET and water yield (R) over a long-term period when the change in soil water storage is negligible. Figure 3 summarizes the changes in total annual P, ET, and R over the NFs with respect to the baseline levels (i.e., 1970–1999). The median values show that annual P is expected to increase by 17 mm yr^−1^ (2%) in 2030s and 42 mm yr^−1^ (5%) in 2080s under RCP4.5, and by 28 mm yr^−1^ (3%) and 51 mm yr^−1^ (6%) at the same time under RCP8.5. The counterparts for change in ET reach 33 (8%), 64 (15%), 41 (10%), and 101 (24%) mm yr^−1^, respectively. It is clear that the magnitudes of increase in ET exceed that of P and in turn lead to a considerable decline in R for all the future scenarios, with the corresponding median reduction of 18 (4%), 23 (5%), 18 (4%), and 31 (7%) mm yr^−1^. Although both positive and negative changes in future P and R have been found, the IQRs remain positive for P and ET, and negative for R, indicating a high level of agreement on the changing directions for all three variables. Furthermore, the results suggest that R may decrease more sharply with larger uncertainty by the second 30-yr period in future (i.e., 2070–2099), reaching a possible maximal decrease up to 24% under RCP8.5.

**Figure 4:**
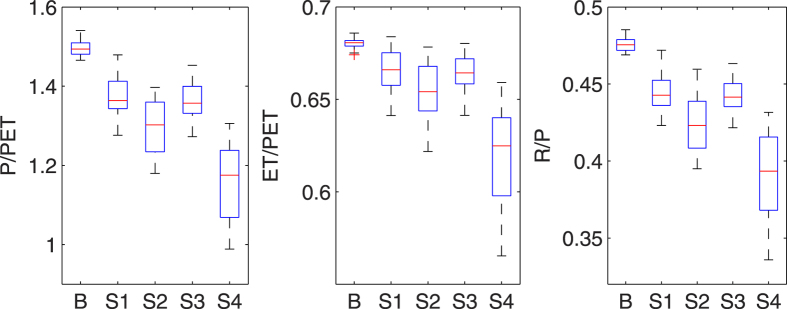
Multi-year average ratios of P/PET, ET/PET and R/P in the baseline and future scenarios for the entire NF area. Vertical position of the boxes in the y-axis displays the range of ratios simulated with outputs of different GCMs, and the five scenarios are denoted by ‘B’ (baseline), ‘S1’ (RCP4.5/2030s), ‘S2’ (RCP4.5/2080s), ‘S3’ (RCP8.5/2030s), and ‘S4’ (RCP8.5/2080s) along the x-axis.

Alterations of water partitioning pattern are further examined by comparing the averaged ratios of P/PET, ET/PET and R/P over the entire NF area under the baseline and future scenarios:

- (i)  P/PET. The ratio of annual precipitation to potential evapotranspiration is a commonly used index of humidity or aridity^27^. The median value of P/PET (Fig. 4) is predicted to decrease from 1.49 (baseline) to 1.36 (RCP4.5/2030s), 1.30 (RCP4.5/2080s), 1.36 (RCP8.5/2030s), and 1.18 (RCP8.5/2080s). A general decreasing trend from the baseline to 2030s and from 2030s to 2080s is found at both emission levels, with the largest decrease in RCP8.5/2080s scenario. Judging from the diminishing P/PET, the NFs may face higher drought risk even though more precipitation can be expected in the future.
- (ii) ET/PET. The ratio of ET/PET is a water stress index indicating the stress level that water supply meets atmospheric evaporative demand. Our study shows that the variation in ET/PET is not as dramatic as P/PET due to the significant rise in ET. The median value of the ratio stays between 0.62 ~ 0.68 across all the scenarios. Nevertheless, there is a clear decreasing trend from the baseline to 2030s and then to 2080s under both RCP4.5 and RCP8.5, suggesting an increase of water stress.
- (iii) R/P. The ratio of R/P represents the portion of precipitation that becomes water yield, which directly affects the water supply for society. Given that forested lands are the most productive source of clean water in the U.S., this ratio is a good indicator of water supply function of the NFs. A decreasing signal is found in the variations of R/P ratio due to the generally larger rise in ET than precipitation in the future. Consistent with the decreasing trends in R, the mean R/P ratio over the whole area is projected to decrease from an IQR of 0.47 ~ 0.48 in the baseline to 0.44 ~ 0.45, 0.41 ~ 0.44, 0.44 ~ 0.45, and 0.37 ~ 0.42 in the four future scenarios, while the median line shifts from 0.48 to 0.44, 0.42, 0.44, and 0.39.

### Changes in ecosystem productivity

**Figure 5:**
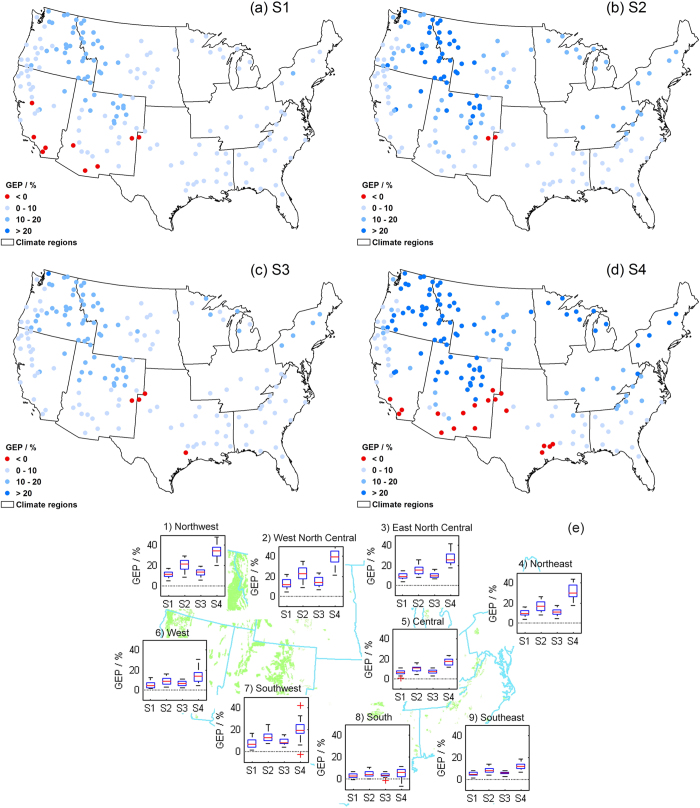
Same as Fig. 2, but for the percent changes in multi-year mean annual GEP (%). Maps were generated using ArcGIS 10.0 (www.esri.com/software/arcgis).

Gross ecosystem productivity (GEP) is projected to increase over most of the NFs (Fig. 5a–d), peaking in NFs around the borders of the Northwest, West North Central and Southwest regions with average increases over 30% and 50% by 2080s under RCP4.5 and RCP8.5, respectively. Decreasing GEP is found only in a small part of the West, Southwest and South regions, with the largest decreases in Kiowa National Grassland (Southwest) and Rita Blanca National Grassland (South) (<5%). The box plots (Fig. 5e) show the consistencies and discrepancies in multi-model estimated GEP for each region. GEP in northern part of the CONUS is projected to increase at a higher level up to approximately 50%, where the variability among models is also more notable. Meanwhile, there is more consistency in the results of NFs located in southern half of the U.S., particularly in the South and Southeast regions, where the IQRs fall between zero and 20%. However, the magnitudes of these relative changes must be evaluated with the absolute GEP change and background values. For example, because the baseline GEP is naturally high in the southeastern U.S., where water and energy supplies are plentiful, a large absolute change in GEP can be rather small in terms of relative change.

**Figure 6:**
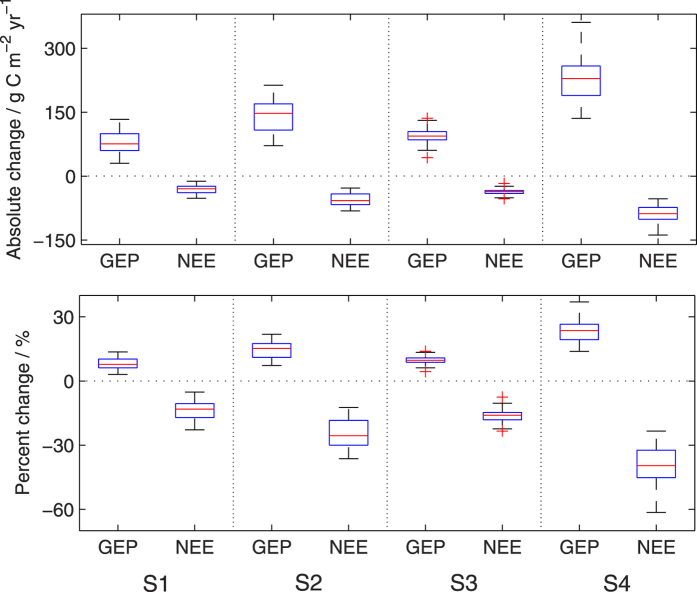
Changes in total GEP and NEE over the NF areas from the baseline to the future scenarios of S1 (RCP4.5/2030s), S2 (RCP4.5/2080s), S3 (RCP8.5/2030s), and S4 (RCP8.5/2080s).

The magnitudes of overall change in annual GEP and NEE vary significantly among models (Fig. 6). The increment of median GEP rises from 76 g C m^−2^ yr^−1^ (8%) in 2030s to 147 g C m^−2^ yr^−1^ (15%) in 2080s under RCP4.5 scenario, and from 94 g C m^−2^ yr^−1^ (10%) in 2030s to 229 g C m^−2^ yr^−1^ (24%) in 2080s under RCP8.5. NEE is the difference between ecosystem respiration (Re) and GEP. When NEE is positive, carbon is released by the ecosystem to the atmosphere (i.e., carbon source); and when NEE is negative, carbon is sequestered from the atmosphere to the ecosystem (i.e., carbon sink). The median changes in NEE reach −30 (−13%), −58 (−26%), −36 (−16%) and −88 (−40%) g C m^−2^ yr^−1^ under the four future scenarios, respectively. The increase in both GEP and absolute NEE jumps significantly from 2030s to 2080s, with the IQRs varying between 19 ~ 70 (2% ~ 7%) and 7 ~ 28 (4% ~ 13%) g C m^−2^ yr^−1^.

### Divergence of changes in ecosystem productivity and water yield

**Figure 7:**
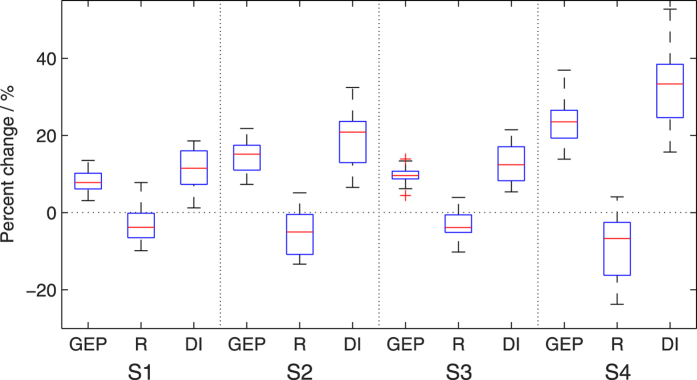
Percent changes (%) in total GEP, R, and the divergence of them (DI) over the NF areas from the baseline to the future scenarios of S1 (RCP4.5/2030s), S2 (RCP4.5/2080s), S3 (RCP8.5/2030s), and S4 (RCP8.5/2080s).

The increasing GEP and decreasing R show a notable divergence in the response to climate change (Fig. 7). The magnitude of divergence (DI) is defined as:


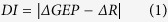


where *ΔGEP* and *ΔR* are the percent changes in GEP and R from the baseline to a future period.

As a result of the opposite changing directions of GEP and R, DI is projected to vary over time and GHG emission level. The median DI increases from RCP4.5/2030s (11.5%) to RCP8.5/2030s (12.4%), RCP4.5/2080s (20.9%), and RCP8.5/2080s (33.4%) scenarios, with the IQR expanding from 8.7% to 8.8%, 10.7%, and 13.8%. The variation patterns are consistent with the projected temperature rise under the future scenarios, indicating that the divergence of GEP and R is likely to be further enhanced by higher GHG emissions, and the prediction uncertainty becomes larger.

## Discussion

We provide a comprehensive assessment on the potential impacts of climate change on key ecosystem services using the most recent CMIP5 datasets and a consistent modeling approach across the 170 NFs of the CONUS. Precipitation and ET fluxes over the entire area are consistently predicted to increase in the two future periods of 2020–2049 and 2070–2099 under the RCP4.5 and RCP8.5 scenarios when compared to the baseline of 1970–1999. In contrast, the variation of water yield shows an opposite trend. Overall ecosystem productivity (GEP) and net carbon sink strength (absolute value of NEE) are both projected to increase significantly. Spatially, more declines in water yield are projected in inland forests than the coastal areas. ET and GEP are projected to increase over most of the NFs, while decreasing trend can be observed occasionally across the West, Southwest and South regions.

Precipitation is a primary control of water yield and ecosystem productivity in the U.S.^3^,^28^,^29^,^30^. Our previous study quantifying the impacts of historical drought at ecosystem functions of the 170 NFs suggests that decrease in precipitation, and thus ET, usually leads to corresponding reduction in both water yield and GEP^26^. However, the current study reveals that future climate change may alter the water partitioning pattern profoundly in NFs by enhancing ET and depressing water yield, in spite of the general increasing trend of precipitation. On the whole, median precipitation is projected to increase by 17 (2%) ~ 51 (6%) mm yr^−1^ under the future scenarios, resulting in an overall meteorological wetting condition in forested lands in the CONUS. However, because of the large rise of air temperature and PET, forest lands are expected to become drier as indicated by decreased P/PET and R/P ratios. The increase in median ET, which reaches 33 (8%) ~ 101 (24%) mm yr^−1^, clearly offsets the increase in precipitation and in turn leads to a considerable decline in water yield. From a long-term perspective, the major threat that climate change poses on forest ecosystem is not decrease in precipitation input, but altering water partitioning pattern in hydrologic cycle.

Due to the close coupling of water and carbon cycling through the ecohydrological processes, changes in water partitioning can be one of the vital factors driving the response of ecosystem productivity to climate change. ET implies the combination of water (i.e., precipitation) and energy (i.e., temperature) availability, and acts as a major control of ecosystem productivity^9^,^12^,^28^. With the shift in water partitioning pattern, as well as the rising temperature and precipitation, the two primary ecosystem services of NFs (i.e., water supply and carbon sequestration) will probably respond to climate change divergently. GEP (median value) is expected to be enhanced by 76 (8%) ~ 229 (24%) g C m^−2^ yr^−1^, at the expense of water yield decreasing by 18 (4%) ~ 31 (7%) mm yr^−1^. The projected maximum changes in GEP and water yield reach 37% and −24%, respectively. Our results also suggest that the divergence of ecosystem services tends to become much larger over time in later part of the 21^st^ century with increasing uncertainty, especially when under a higher GHG emission scenario (RCP8.5).

Uncertainties should be recognized when interpreting the results from this modeling study. The varying magnitudes and directions of future climate change projected by various GCMs indicate the significant uncertainties in the state-of-art climate models. The diverse biases of the chosen GCMs may affect the downscaling of monthly meteorological variables and compound the results with the uncertainty from downscaling model structure^31^,^32^. Moreover, the WaSSI ecosystem model was parameterized with limited global eddy flux data that do not cover all ecosystem types in the U.S., especially those found in mountainous regions. Thus uncertainties from model parameters may also affect our results. The uncertainty of WaSSI model structure is another possible uncertainty source. WaSSI uses an empirical relationship between carbon and water derived from direct observations from eddy flux sites, and it does not describe the detailed physical processes of carbon fluxes. The equation for estimating ecosystem ET from precipitation, LAI, and PET was fixed for each land cover type. The water use efficiency (i.e., WUE = GEP/ET) for a specific plant functional type was also set as a constant. Several other factors that may affect water use efficiency were not accounted for, such as changes in the land use, atmospheric background (e.g., carbon and nitrogen concentrations), and the impact of high temperature on photosynthesis^28^,^33^. Such simplifications of ecohydrological processes are essential for long-term, continental scale modeling, but the model might not capture subtle process-level interactions or the extreme climate impacts on forest ecosystem.

The current modeling study focuses on water-carbon interactions and essentially examines the sensitivity of GEP and water yield to future climate perturbations. The projections of GEP are based on the fundamental relationships between ET and GEP across biomes^12^. However, forest ecosystem responses to future climate can be rather complex and multi-directional. It remains a challenge to realistically quantify the response of regional forest GEP to climate change under multiple environmental stressors^29^,^30^,^34^. At the stand level, different models may produce quite different results because of the diverse regional environmental stresses, such as soil nutrient level, species composition, atmospheric deposition (e.g., sulfur and nitrogen), and air pollutants (e.g., CO_2_, ozone)^28^,^34^,^35^,^36^. In addition, current ecosystem models do not have the capacity to fully simulate the vegetation response to climate change^37^, which may substantially increase the range of uncertainty. Given these caveats, we can conclude that our results generally agree with previous projections of the U.S. forest carbon balance: future climates with increased precipitation and temperature with higher CO_2_ concentration are likely to enhance ecosystem productivity at global or continental scales^10^,^30^.

In spite of the uncertainties identified, this work provides an important step toward comprehensively assessing climate change impacts on ecosystem services for NFs in the CONUS. Understanding the tradeoffs and divergent response of ecosystem service components at the regional and continental scales is critical for land management under climate change. The study provides a valuable reference for land/water management and adaptation planning in NFs. Our modeling results can help to prioritize watersheds in NFs to maximize ecosystem services while minimizing negative impacts of the potential climate change^38^. For example, an increase of ecosystem productivity in some NFs may result in an increasing forest biomass (i.e., forest densification) and fuel loading and thus wildfire risks^39^,^40^. A drying trend in these forest watersheds threatens water supply^4^ and other water-related ecosystem services (e.g., aquatic habitats), and forests themselves^38^. Active forest management practices such as thinning, selective cutting, and prescribed burning may be implemented to reduce fire risk and increase forest resiliency to droughts and climate change^3^,^38^.

## Methods

### Study area

The study area is the 170 NFs that cover around 688,000 km^2^ across the CONUS, which can be divided into nine climatically-consistent regions according to historical analysis, including Northwest (R01), West North Central (R02), East North Central (R03), Northeast (R04), Central (R05), West (R06), Southwest (R07), South (R08) and Southeast (R09)^41^. More than half of the NF areas (52%) are located in the Northwest and Southwest regions, and another 33% areas lie in the West North Central and West regions (Table S2). The observed historical data (i.e., 1962–2012) 25,26 suggest that the NFs span a large climatic gradient, with the multi-year mean annual precipitation and temperature ranging from 299 mm (Crooked River National Grassland) and 0.8 °C (Bridger National Forest) to 2, 989 mm (Olympic National Forest) and 21.3 °C (Ocala National Forest) (Table S3).

### Climate data

The raw output of GCM simulations are statistically downscaled using the Multivariate Adaptive Constructed Analogs (MACA) method^42^ to provide higher spatial resolution output than the coarser GCMs, with the LIVNEH^43^ observational dataset as training data. This method allows global output to be translated to local and regional projections using statistical relationships between climate variables. The datasets of downscaled output for the CONUS (the MACAv2-LIVNEH) and more information of the used GCMs can be acquired from http://maca.northwestknowledge.net/.

### Ecohydrological modeling

The WaSSI model is an integrated water-centric monthly model that simulates water, energy and carbon cycles at a watershed scale. The extensive validations with USGS gauged streamflow^44^,^45^, global eddy fluxes monitoring and remote sensing products of ET and GEP^12^,^25^ suggest that WaSSI can achieve an acceptable modeling accuracy with respect of both water and carbon fluxes at broad spatial scales. WaSSI performs water and carbon simulations with several mathematical sub-models. A conceptual snow sub-model^46^ is supplemented to approximate the partitioning of precipitation into rainfall and snowfall, as well as the processes of snowpack melt and accumulation. The ecosystem ET model^11^,^12^ estimates ET as a function of precipitation, leaf area index (LAI), and Hamon potential evapotranspiration (PET) for each land cover type within the watershed. Actual ET is then calculated by constraining this estimated value with soil water availability. Hydrologic processes including infiltration, soil moisture storage and water yield are simulated using algorithms of the Sacramento Soil Moisture Accounting model (SAC-SMA)^47^. GEP, NEE, and Re as indicators of carbon sequestration are modeled based on the empirical statistics between GEP and ET by land cover types. Re is estimated by a set of linear regression equations as a function of GEP. NEE is modeled as the difference between GEP and Re (Re–GEP) at a monthly scale. More details of model development and validation can be found in the previous publications^12^,^25^,^44^.

## Additional Information

**How to cite this article**: Duan, K. *et al.* Divergence of ecosystem services in U.S. National Forests and Grasslands under a changing climate. *Sci. Rep.* **6**, 24441; doi: 10.1038/srep24441 (2016).

## Supplementary Material

Supplementary Information
