# Supplementary material for: Divergence of ecosystem services in U.S. National Forests and Grasslands under a changing climate
Source: Sci Rep. 2016 Apr 21;6:24441. doi: 10.1038/srep24441 (PMC4839213; doi:10.1038/srep24441)
Supplement: Supplementary Information [file srep24441-s1.pdf]

Divergence of ecosystem services in U.S. National Forests and Grasslands under a changing climate

Kai Duan[a], Ge Sun[b*], Shanlei Sun[c], Peter V. Caldwell[d], Erika C. Cohen[b], Steven G. McNulty[b], Heather D. Aldridge[e], Yang Zhang[a]

[a] *Department of Marine, Earth, and Atmospheric Sciences, North Carolina State University, Raleigh, NC, USA*

[b] *Eastern Forest Environmental Threat Assessment Center, USDA Forest Service, Raleigh, NC, USA*

[c] *Key Laboratory of Meteorological Disaster of Ministry of Education, Nanjing University of Information Science & Technology, Nanjing, Jiangsu, China*

[d] *Coweeta Hydrologic Laboratory, USDA Forest Service, Otto, NC, USA*

[e] *State Climate Office of North Carolina, North Carolina State University, Raleigh, NC, USA*

Correspondence to G. Sun (email: gesun@fs.fed.us)

Supplementary Table S1. Average changes in mean annual precipitation and temperature over the 170 NFs from the baseline scenario (B) to future scenarios (S1~S4)

Model	Country	Precipitation / mm yr^{-1}					Temperature / ℃				
		B	S1	S2	S3	S4	B	S1	S2	S3	S4
bcc-csm1-1	China	892	-2	-10	+20	-11	6.2	+1.6	+2.5	+2.0	+4.9
bcc-csm1-1-m	China	887	+22	-18	+47	+49	6.2	+1.3	+2.5	+1.7	+4.4
BNU-ESM	China	911	+62	+80	+28	+103	6.2	+2.0	+3.4	+2.1	+5.6
CanESM2	Canada	902	+45	+90	+44	+187	6.1	+2.4	+3.8	+2.6	+6.2
CCSM4	USA	890	+17	+62	+35	+67	6.3	+1.5	+2.6	+2.0	+4.6
CNRM-CM5	France	883	+76	+93	+61	+130	6.2	+1.4	+2.8	+1.6	+4.8
CSIRO-Mk3-6-0	Australia	883	-2	+50	+19	+81	5.9	+1.8	+3.5	+2.0	+5.6
GFDL-ESM2M	USA	894	+22	+42	+56	+65	6.0	+1.6	+2.3	+1.7	+4.4
GFDL-ESM2G	USA	892	+47	+36	+28	+32	6.1	+1.1	+1.7	+1.3	+3.7
HadGEM2-ES	UK	887	+33	+37	+34	+42	6.1	+1.9	+3.5	+2.3	+6.6
HadGEM2-CC	UK	884	-10	+30	+22	+23	6.1	+2.1	+4.0	+2.4	+6.5
inmcm4	Russia	875	+17	+13	-5	+51	6.2	+0.8	+1.7	+1.1	+3.5
IPSL-CM5A-LR	France	892	+14	+2	-16	+51	6.2	+1.9	+3.2	+1.9	+6.1
IPSL-CM5A-MR	France	900	+3	+43	+9	-18	6.0	+1.9	+3.3	+2.4	+6.1
IPSL-CM5B-LR	France	890	+21	+61	+38	+87	6.1	+1.5	+2.3	+1.7	+4.3
MIROC5	Japan	900	+15	+8	+20	+27	5.8	+2.3	+3.6	+2.4	+5.4
MIROC-ESM	Japan	903	+34	+26	+18	+34	6.1	+2.4	+4.3	+2.6	+6.7
MIROC-ESM-CHEM	Japan	888	+16	+42	+38	+14	6.1	+2.4	+4.2	+2.8	+7.0
MRI-CGCM3	Japan	889	+2	+49	+25	+83	6.2	+0.8	+1.8	+1.0	+3.3
NorESM1-M	Norway	891	-8	+61	+36	+100	6.1	+1.7	+3.1	+2.1	+5.0

Supplementary Table S2. Summary of the distribution of the 170 NFs in the nine climate regions

NO.	Region	NFs covered	Area / 10^3 km^2	Multi-year mean (min ~ max) during 1962-2012	
				Precipitation / mm yr^{-1}	Temperature / ℃
1	Northwest	41	183 (26.6%)	991 (299~2989)	5.8 (1.2~11.6)
2	West North Central	32	121 (17.6%)	766 (338~1283)	3.9 (0.8~8.9)
3	East North Central	8	29 (4.2%)	816 (680~1323)	4.6 (2.9~8.3)
4	Northeast	4	7 (1.0%)	1157 (811~1352)	5.6 (4.6~8.9)
5	Central	11	18 (2.7%)	1218 (1046~1655)	11.9 (8.9~16.4)
6	West	25	107 (15.6)	934 (301~2346)	9.1 (6.4~15.3)
7	Southwest	37	174 (25.4%)	547 (353~788)	7.4 (2.0~15.5)
8	South	22	23 (3.3%)	1321 (364~1564)	16.1 (11.4~19.6)
9	Southeast	19	25 (3.7%)	1340 (1046~1655)	15.2 (8.9~21.3)

Supplementary Table S3. Watershed area and multi-year means of annual precipitation and temperature during 1962-2012 for the 170 National Forests and Grasslands

NO.	Name	Area/km^2	Precipitation /mm yr^{-1}	Temperature/^{o}C
1	Sam Houston National Forest	660.7	1193	19.6
2	Los Padres National Forest	1372.6	755	13.5
3	Cleveland National Forest	1718.5	499	15.3
4	San Bernardino National Forest	838.8	441	15.0
5	Angeles National Forest	10339.4	583	13.8
6	Sequoia National Forest	30425.9	792	7.6
7	Prescott National Forest	2707.1	462	13.0
8	Toiyabe National Forest	9965.2	301	8.0
9	Cibola National Forest	6535.6	353	9.9
10	Coronado National Forest	6956.2	464	15.5
11	Lincoln National Forest	4438.6	483	11.4
12	Gila National Forest	641.5	385	13.2
13	Caddo National Grassland	71.4	1102	17.3
14	Apache National Forest	0.3	560	8.9
15	Tonto National Forest	2.2	532	14.1
16	Coconino National Forest	70.8	561	10.0
17	Lyndon B. Johnson National Grassland	81.4	876	17.7
18	Sitgreaves National Forest	44494.8	504	11.8
19	Santa Fe National Forest	771.5	384	9.6
20	Black Kettle National Grassland	126.5	603	14.8
21	Kaibab National Forest	3978.6	433	9.7
22	Kiowa National Grassland	518.0	382	11.3
23	McClellan Creek National Grassland	5.7	574	14.6
24	Comanche National Grassland	1795.8	364	11.9
25	Carson National Forest	4932.4	575	5.7
26	Rita Blanca National Grassland	412.8	392	12.6
27	San Juan National Forest	71.9	566	2.0
28	Rio Grande National Forest	2.6	545	6.5
29	Cimarron National Grassland	439.6	422	13.0
30	San Isabel National Forest	1137.6	550	6.5
31	Uncompahgre National Forest	2317.5	547	7.2
32	Gunnison National Forest	19.8	410	2.0
33	De Soto National Forest	2158.9	1564	18.7
34	Kisatchie National Forest	2461.6	1425	18.7
35	Angelina National Forest	641.7	1300	18.8
36	Davy Crockett National Forest	652.7	1153	19.1
37	Sabine National Forest	652.7	1311	18.4
38	Ocala National Forest	1553.1	1285	21.3
39	Apalachicola National Forest	2319.9	1332	19.9
40	Osceola National Forest	940.5	1552	19.4

41	Conecuh National Forest	339.9	1537	18.4
42	William B. Bankhead National Forest	736.5	1462	15.6
43	Sumter National Forest	1167.1	1443	15.9
44	Chattahoochee National Forest	5947.7	1655	13.2
45	Holly Springs National Forest	631.8	1356	15.7
46	Ouachita National Forest	7226.4	1170	16.2
47	Homochitto National Forest	777.3	1507	18.4
48	Tuskegee National Forest	45.9	1340	17.4
49	Bienville National Forest	729.5	1458	17.5
50	Talladega National Forest	1588.5	1396	16.3
51	Delta National Forest	251.5	1398	17.7
52	Oconee National Forest	470.9	1191	17.1
53	Tombigbee National Forest	272.1	1422	16.6
54	Francis Marion National Forest	1049.9	1285	18.0
55	Pisgah National Forest	1785.0	1303	16.4
56	Ozark National Forest	4600.3	1563	11.8
57	St. Francis National Forest	86.1	1249	14.7
58	Nantahala National Forest	639.0	1140	15.4
59	Uwharrie National Forest	206.7	1587	12.2
60	Cherokee National Forest	2426.1	1203	11.6
61	Shawnee National Forest	1134.4	1250	12.7
62	Jefferson National Forest	1783.8	1130	13.2
63	Daniel Boone National Forest	2282.4	1103	11.6
64	Mark Twain National Forest	6088.5	1181	13.7
65	Hoosier National Forest	821.3	1179	12.5
66	Croatan National Forest	652.5	1399	16.9
67	Sierra National Forest	35.2	829	12.6
68	Inyo National Forest	4.9	395	7.4
69	Dixie National Forest	7630.4	461	6.5
70	Stanislaus National Forest	95.7	1224	11.4
71	Humboldt National Forest	9713.1	450	6.4
72	Eldorado National Forest	93.4	1362	11.8
73	Tahoe National Forest	11289.3	1152	8.3
74	Mendocino National Forest	73.8	1255	12.4
75	Lassen National Forest	589.4	1281	11.2
76	Plumas National Forest	127.8	1450	11.6
77	Klamath National Forest	1330.0	877	9.5
78	Shasta National Forest	1897.0	1605	11.9
79	Trinity National Forest	23101.6	1756	11.0
80	Six Rivers National Forest	36.1	1812	10.6
81	Modoc National Forest	40655.9	1100	7.2
82	Butte Valley National Grassland	78.9	434	7.8
83	Siskiyou National Forest	44.5	1083	7.5
84	Rogue River National Forest	25.9	2346	11.6

85	Boise National Forest	306.5	680	7.8
86	Fremont National Forest	408.2	509	6.9
87	Winema National Forest	15.3	1008	5.9
88	Umpqua National Forest	258.2	1199	10.9
89	Sawtooth National Forest	2436.1	464	7.0
90	Manti-La Sal National Forest	7243.6	514	5.8
91	Fishlake National Forest	3777.5	485	6.7
92	White River National Forest	11.5	639	2.1
93	Pike National Forest	358.5	566	3.6
94	Grand Mesa National Forest	41028.4	618	3.3
95	Arapaho National Forest	27.4	543	3.0
96	Uinta National Forest	1154.4	483	7.2
97	Roosevelt National Forest	6857.1	565	2.9
98	Wasatch National Forest	11662.3	649	3.5
99	Medicine Bow National Forest	11748.6	706	3.0
100	Pawnee National Grassland	780.8	355	8.8
101	Routt National Forest	3650.4	788	2.8
102	Nebraska National Forest	569.9	515	8.8
103	Cache National Forest	2836.3	759	4.9
104	Thunder Basin National Grassland	2237.6	338	7.6
105	Teton National Forest	4.15	719	2.6
106	Caribou National Forest	1080.8	581	6.9
107	Bridger National Forest	0.2	575	0.8
108	Targhee National Forest	1.8	536	1.2
109	Curlew National Grassland	192.2	448	7.6
110	Challis National Forest	1689.3	496	3.1
111	Custer National Forest	2734.1	393	7.0
112	Salmon National Forest	2467.8	500	2.3
113	Shoshone National Forest	0.3	498	3.2
114	Beaverhead National Forest	120861.8	842	3.1
115	Gallatin National Forest	1174.2	733	3.4
116	Bighorn National Forest	4471.2	629	2.2
117	Buffalo Gap National Grassland	1929.1	426	8.9
118	Black Hills National Forest	5058.8	555	5.8
119	Oglala National Grassland	829.9	516	8.8
120	Samuel R. McKelvie National Forest	468.8	409	8.2
121	Grand River National Grassland	626.1	420	6.7
122	Fort Pierre National Grassland	468.5	469	8.5
123	Deschutes National Forest	35.5	2219	11.2
124	Siuslaw National Forest	2535.6	614	6.4
125	Willamette National Forest	44.1	2054	8.7
126	Gifford Pinchot National Forest	25557.2	1344	9.9
127	Mt. Hood National Forest	50.2	1715	5.1
128	Malheur National Forest	14613.9	654	6.0

129	Whitman National Forest	9421.4	805	6.0
130	Umatilla National Forest	1111.0	537	6.9
131	Ochoco National Forest	2483.3	508	6.6
132	Wallowa National Forest	11.1	537	6.9
133	Crooked River National Grassland	453.3	299	8.4
134	Payette National Forest	295.1	768	6.1
135	Nezperce National Forest	1.8	644	7.3
136	St. Joe National Forest	710.9	1029	6.3
137	Snoqualmie National Forest	473.9	1816	6.9
138	Wenatchee National Forest	116.2	716	7.4
139	Clearwater National Forest	88.3	1283	3.5
140	Olympic National Forest	2559.7	2989	8.3
141	Coeur D Alene National Forest	8.5	934	6.9
142	Kaniksu National Forest	12522.1	1006	5.3
143	Mt. Baker National Forest	1846.3	2638	6.4
144	Okanogan National Forest	2768.2	525	5.3
145	Colville National Forest	390.6	644	6.5
146	Deerlodge National Forest	3280.6	533	3.9
147	Lolo National Forest	1901.2	715	5.2
148	Cedar River National Grassland	27.2	420	6.2
149	Little Missouri National Grassland	4149.9	512	4.5
150	Helena National Forest	1291.9	383	5.9
151	Sheyenne National Grassland	284.4	537	5.5
152	Lewis & Clark National Forest	17512.8	931	3.5
153	Bitterroot National Forest	11.2	483	5.0
154	Kootenai National Forest	334.4	847	3.8
155	Flathead National Forest	4452.8	603	4.8
156	George Washington National Forest	4616.6	1046	10.8
157	Monongahela National Forest	4005.7	1274	8.9
158	Wayne National Forest	987.3	1060	11.4
159	Allegheny National Forest	2079.0	1136	7.3
160	Manistee National Forest	2180.3	879	8.3
161	Finger Lakes National Forest	66.2	856	7.3
162	Nicolet National Forest	1229.3	826	4.8
163	Huron National Forest	1779.2	795	5.2
164	Chequamegon National Forest	3485.6	753	6.6
165	Green Mountain National Forest	1620.9	811	5.3
166	Hiawatha National Forest	3578.3	680	3.8
167	Chippewa National Forest	2718.8	1323	5.4
168	Ottawa National Forest	5475.6	802	4.4
169	Superior National Forest	8665.9	735	2.9
170	White Mountain National Forest	3189.7	1352	4.6
